# Unlocking the Secrets of Adipose Tissue: How an Obesity-Associated Secretome Promotes Osteoblast Dedifferentiation via TGF-β1 Signaling, Paving the Path to an Adipogenic Phenotype

**DOI:** 10.3390/cells13171418

**Published:** 2024-08-25

**Authors:** Yasmin Silva Forte, Vany Nascimento-Silva, Caio Andrade-Santos, Isadora Ramos-Andrade, Georgia Correa Atella, Luiz Guilherme Kraemer-Aguiar, Paulo Roberto Falcão Leal, Mariana Renovato-Martins, Christina Barja-Fidalgo

**Affiliations:** 1 Laboratory of Cellular & Molecular Pharmacology, Department of Cell Biology, Instituto de Biologia Roberto Alcantara Gomes (IBRAG), Universidade do Estado do Rio de Janeiro, Rio de Janeiro 20551-030, Brazil; yasmin.forte@gmail.com (Y.S.F.); vanysilva@hotmail.com (V.N.-S.); caio.andrade2703@gmail.com (C.A.-S.); isadorar.andrade@yahoo.com.br (I.R.-A.); 2 Institute of Medical Biochemistry, Universidade Federal do Rio de Janeiro, Rio de Janeiro 21941-902, Brazil; atella@bioqmed.ufrj.br; 3 Obesity Unit, Multiuser Clinical Research Center (CePEM), Hospital Universitário Pedro Ernesto, Universidade do Estado do Rio de Janeiro, Rio de Janeiro 20551-030, Brazil; lgkraemeraguiar@gmail.com (L.G.K.-A.); prfalcaoleal@gmail.com (P.R.F.L.); 4 Department of Molecular & Cellular Biology, Universidade Federal Fluminense, Rio de Janeiro 24020-141, Brazil; m_renovatomartins@yahoo.com.br

**Keywords:** obesity, adipose tissue, osteogenesis, adipogenesis, dedifferentiation, TGF-β1

## Abstract

Background: Obesity poses a significant global health challenge, given its association with the excessive accumulation of adipose tissue (AT) and various systemic disruptions. Within the adipose microenvironment, expansion and enrichment with immune cells trigger the release of inflammatory mediators and growth factors, which can disrupt tissues, including bones. While obesity’s contribution to bone loss is well established, the direct impact of obese AT on osteoblast maturation remains uncertain. This study aimed to explore the influence of the secretomes from obese and lean AT on osteoblast differentiation and activity. Methods: SAOS-2 cells were exposed to the secretomes obtained by culturing human subcutaneous AT from individuals with obesity (OATS) or lean patients, and their effects on osteoblasts were evaluated. Results: In the presence of the OATS, mature osteoblasts underwent dedifferentiation, showing an increased proliferation accompanied by a morphological shift towards a mesenchymal phenotype, with detrimental effects on osteogenic markers and the calcification capacity. Concurrently, the OATS promoted the expression of mesenchymal and adipogenic markers, inducing the formation of cytoplasmic lipid droplets in SAOS-2 cells exposed to an adipogenic differentiation medium. Additionally, TGF-β1 emerged as a key mediator of these effects, as the OATS was enriched with this growth factor. Conclusions: Our findings demonstrate that obese subcutaneous AT promotes the dedifferentiation of osteoblasts and increases the adipogenic profile in these cells.

## 1. Introduction

Obesity is a complex and widespread chronic disease with pandemic proportions. Its high prevalence raises alarms due to its strong association with the onset and progression of various other health disorders, including cardiovascular diseases, several types of cancer, diabetes, and liver and kidney diseases, as well as a direct link with increased mortality [1]. The development of obesity is influenced by a combination of genetic, environmental, and behavioral factors, ultimately resulting in an imbalance between caloric intake and energy expenditure. This imbalance leads to the expansion of adipose tissue (AT), primarily through the hypertrophy and/or hyperplasia of adipocytes, which accumulate triglycerides [2]. Under these stress conditions, obese AT starts expressing and releasing cytokines such as TNF-α, IL-8, IL-6, leptin, resistin, monocyte chemoattractant protein-1 (MCP-1), and TGF-β, indicating the establishment of chronic low-grade inflammation. These adipokines enter the bloodstream and disrupt the homeostasis of various organs and tissues, including bone tissue [3].

The bone tissue is a specialized connective tissue with a mineralized extracellular matrix. Aside from forming the skeletal system and supporting the structure and locomotion of mammals, bones serve as mineral and fat storage, produce hormones, and play a role in hematopoiesis and vital organ protection [4]. Bone tissue continuously undergoes renewal and remodeling processes, which rely on the balanced activity of osteoblasts (bone-forming cells) and osteoclasts (bone-resorbing cells). Imbalances in this delicate equilibrium can lead to conditions such as osteoporosis, which is characterized by bone mass loss, or osteopetrosis, which is characterized by excessive bone density [5].

Osteoprogenitor cells with a high level of mitotic activity, a fibroblastic morphology, and the expression of Runx2 differentiate from mesenchymal stem cells (MSCs) in a microenvironment rich in growth and differentiation factors, transitioning into osteoblast cells. During prolonged exposure to osteogenic differentiation factors, osteoblasts upregulate initial markers such as type I collagen (COLAI) and alkaline phosphatase (ALP), followed by late markers such as osteopontin (OPN), osteocalcin (OCN), and bone sialoprotein (BSP). Mature osteoblasts exhibit a cuboidal phenotype, limited proliferation, and the ability to promote the calcification of the bone extracellular matrix [6].

The impact of obesity on bone remodeling is a multifaceted and intricate matter influenced by various factors, including gender, age, the site of adipose and bone tissue, the hormonal and metabolic status, and adipokines systemically secreted by obese AT [7]. While some studies have suggested a positive correlation between body mass index (BMI) and bone mineral density (BMD) [8,9,10,11], others have demonstrated an increased risk of fractures and a decreased bone quality associated with a higher BMI and a greater percentage of fat mass [12,13,14]. In this context, the secretome of obese adipose tissue, which is rich in pro-inflammatory adipokines, can induce metabolic disturbances such as insulin resistance, oxidative stress, and alterations in the gut microbiota composition, directly affecting bone remodeling and unbalancing the activities of osteoblasts and osteoclasts, ultimately leading to bone loss [15].

While obesity is known to favor adipogenesis over osteogenesis and to impair osteoblast function through its secretome [15], the idea that it might also induce osteoblast dedifferentiation is a compelling hypothesis [16,17]. It suggests a new avenue for research that could deepen our understanding of how obesity contributes to bone loss and osteoporosis, potentially leading to novel therapeutic strategies to mitigate these effects.

Despite the wealth of data on the influence of obesity on bone remodeling, the direct impact of an obese adipose tissue secretome (OATS) on mature osteoblast differentiation and, consequently, bone formation remains unclear. In a novel approach, our study aimed to investigate the contributions of an OATS and a lean adipose tissue secretome (LATS) on the human osteoblast phenotype and function in vitro. We present the first evidence that secretions from obese adipose tissue impair osteoblast maturation, inducing the regression of mature osteoblasts toward an osteoprogenitor cell phenotype, primarily through TGF-β-mediated signaling. We also observed that dedifferentiated osteoblasts presented an increased adipogenic phenotype.

## 2. Materials and Methods

### 2.1. Participants

Subcutaneous adipose tissue samples were collected from two distinct groups of individuals (Table 1). The experimental group was composed of patients with obesity who underwent bariatric surgery, whose inclusion criterion was a BMI value greater than or equal to 30 kg/m^2^. The exclusion criterion was the non-use of samples from patients with clinically diagnosed diabetes. Adipose tissue samples were obtained during a Roux-en-Y gastric bypass (RYGB) bariatric surgery, performed by minimally invasive (laparoscopic) access. The subcutaneous tissue was removed through the 12 mm portal incision to introduce access to the first portal of the optical camera. The second group included lean individuals undergoing elective plastic surgery with a BMI of 18.5 to 25 kg/m² as the inclusion criteria. The presence of any comorbidities was the exclusion criterion used throughout the study. Samples of subcutaneous adipose tissue from lean patients were collected from the adipose layer on the upper abdominal muscle region in an open surgical field. This study was conducted as a single-blind protocol. None of the patients who agreed to take part in the research were lost during the study and no data were missing. Attrition bias did not occur in this work. Patient recruitment took place at two different medical institutions: the Pedro Ernesto University Hospital (HUPE), with ethical committee approval under CAAE 5602121.2.0000.5259 (approved in 2022), and the Federal Hospital of Ipanema (HFI), with CAAE 36880914.0.0000.5259 (approved in 2019). Both groups comprised female and male patients between the ages of 26 and 45 years. Randomization did not apply to this study.

### 2.2. Adipose Tissue Secretome

**Scheme 1:**
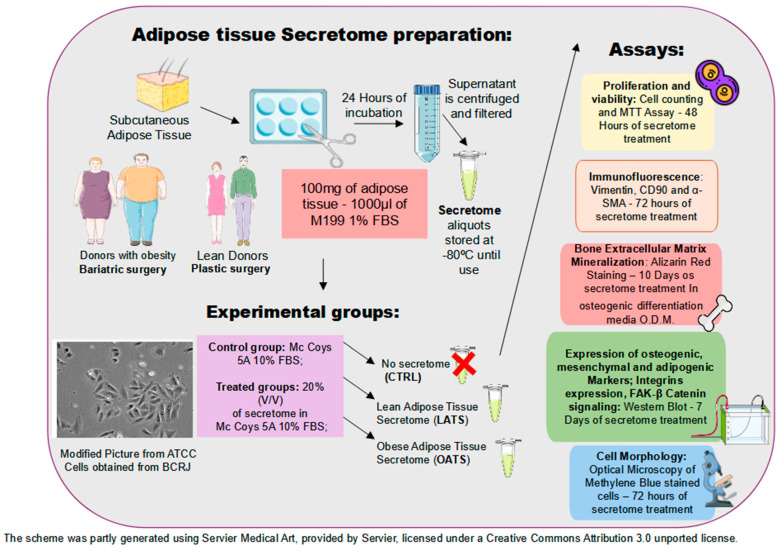
Adipose tissue secretome preparation and experimental design. After adipose tissue secretome obtainment, SAOS-2 cells were treated with the lean adipose tissue secretome (LATS), the obese adipose tissue secretome (OATS), or no secretome (CTRL). Proliferation and viability assays, immunofluorescence, alizarin red staining, a Western blot, and a cell morphology analysis were performed at specific time points.

The secretome derived from cultures of adipose tissue (AT) biopsies from both obese and lean individuals was obtained as previously described [18]. Briefly, subcutaneous AT fragments excised during surgical procedures were rinsed and cleaned with 1× phosphate-buffered saline (1× PBS). Explants weighing 100 mg from AT were then sectioned into small pieces and incubated in 1 mL of 199 medium (M199) (Cat# M3769; Sigma-Aldrich, St. Louis, MO, USA) supplemented with 1% FBS at 37 °C for 24 h. Subsequently, the secretome was collected, followed by centrifugation at 2000× g for 10 min at 4 °C to remove cells and debris. The resulting supernatant was filtered, aliquoted, and stored at −80 °C until further use. A total protein analysis of each secretome (Supplementary Figure S2A) was performed with a Pierce BCA Protein Kit, (Thermo Fisher Scientific, Rockfield, IL, USA). The adipose tissue secretome preparation and experimental design are illustrated in Scheme 1.

### 2.3. Cells and Culture

The human osteoblast cell lineage SaOS-2 (SaOS-2, RRID:CVCL_0548), derived from an immortalized lineage of osteosarcoma, was purchased from the Rio de Janeiro Cell Bank (BCRJ, RJ, Brazil) (BCRJ code 0217). Cell line authentication was performed by a short tandem repeat (STR) DNA genotype analysis on the cells provided by the BCRJ. The cells were cultured in McCoy’s-5A medium (Cat#M4892; Sigma-Aldrich, St. Louis, MO, USA) supplemented with l-glutamine (1.5 mM), sodium bicarbonate (2.2 g/L), HEPES (5.2 g/L), penicillin (60 mg/L), and streptomycin sodium (100 mg/L) until reaching 60–70% confluency. The culture medium was also enriched with 10% fetal bovine serum (FBS). For the experimental procedures, cells from passages P3 to P8 were gently washed with HANK’s/EDTA (5 mM) and subsequently detached using a 0.25% trypsin treatment at 37 °C for 5 min. After washing with fresh media, the cells were seeded in culture bottles or multiwell plates for the specific assays. Human recombinant TGF-β1 (hrTGF-β1) (Cat#100-21; PEPROTECH, Rocky Hill, NJ, USA) and a TGF-βR antagonist (LY2109761: Cat#SML2051; Sigma-Aldrich, St. Louis, MO, USA) were used.

### 2.4. Viability and Proliferation Assays

SAOS-2 cells (2 × 10^4^ cells/well, 6.25 × 10^4^ cells/cm^2^) were seeded onto 96-well plates in McCoy’s-5A medium with 10% FBS for 24 h. After that, the cells were incubated in a fresh medium containing the LATS or the OATS (20% *v*/*v*) or hrTGF-β1 (20–40 ng/mL) for 48 h. Cell viability was evaluated by an MTT assay (Cat#M2128; Sigma-Aldrich, St. Louis, MO, USA) as described previously, with some modifications [19]. To evaluate cell proliferation, SaOS-2 cells (SaOS-2, RRID:CVCL_0548) (1 × 10^4^ cells/well, 3.125 × 10^4^ cells/cm^2^) treated with the LATS, the OATS, or hrTGF-β for 48 h were fixed with ice-cold 96% ethanol and treated with DAPI. DAPI-positive cell nuclei were counted by analyzing images taken under a microscope (Model Olympus IX71 Olympus, Tokyo, Japan) equipped for epifluorescence, using IMAGEJ’s (ImageJ version 1.53p, RRID:SCR_003070) cell counter plugin. The cells from three fields (200×) of each biological replicate were counted, and the average was measured.

### 2.5. Morphology Assessment

SAOS-2 cells (1 × 10^4^ cells/well, 3.125 × 10^4^ cells/cm^2^) were cultured in 96-well plates for three days before treatment with the LATS, the OATS, or hrTGF-β for 72 h. After that, the cells were washed with 1× PBS (pH = 7.4, 37 °C), fixed with ice-cold 95% ethanol for 10 min, washed twice with deionized water, and stained with a 0.2% methylene blue solution in 1× PBS for 5 min, as described previously [20]. After another three washes with deionized water, images were obtained with a microscope (Olympus IX71, Tokyo, Japan) at 200× magnification.

### 2.6. Alizarin Red Staining

After that, to evaluate the extracellular matrix calcification, SAOS-2 cells (1.5 × 10^4^ cells/well, 4.7 × 10^4^ cells/cm^2^), seeded onto 96-well plates for 3 days, were incubated with an osteogenic differentiation medium (McCoy’s 5A with 10% FBS, 10 nM sodium β-glycerophosphate, 50 μg/mL of ascorbic acid, and 1 μM dexamethasone), either without an adipose tissue secretome treatment (as O.D.M.) or in the presence of 20% OATS, LATS, or hrTGF-β for 10 days. The culture medium was renewed every 3–4 days. Cells incubated only with McCoy’s-5A medium with 10% FBS were used as the control. After the treatment, the cells were stained with alizarin red (Cat#A5533; Sigma-Aldrich, St. Louis, MO, USA) to evaluate the matrix calcification, using a protocol described previously [20]. Images of the cells showing matrix calcification were obtained under a microscope at 40× magnification (Olympus IX-71, Tokyo, Japan). To quantify the calcified area, the stain was dissolved in 10% cetylpyridinium chloride (Cat#C0732; Sigma-Aldrich, St. Louis, MO, USA) in 10 mM sodium phosphate (pH = 7), and the absorbance values were measured at 562 nm [21] using an Envision plate reader (Perkin Elmer, Waltham, MA, USA).

### 2.7. Protein Extraction and Western Blotting

Three days after seeding onto 6-well plates (1 × 10^6^ cells/well, 1.06 × 10^5^ cells/cm^2^), the SAOS-2 cells were treated with the LATS, the OATS, or hrTGF-β for a subsequent 7 days, with a medium change after the first 3 days of incubation. After that, the cells were lysed with the RIPA lysis buffer (Cat#R0278; Sigma-Aldrich, St. Louis, MO, USA), and immunoblotting was performed as previously described [18]. Briefly, after denaturation, the proteins were separated using polyacrylamide SDS-PAGE gels (8–12%) and then transferred (Transfer System, BIO-RAD, Hercules, CA, USA) onto a PVDF membrane. After the transfer, the membranes were stained with 0.2% ponceau red to confirm the transference, washed with distilled water, and blocked with 5% skim milk or 5% BSA (Cat#A2153; Thermo Fisher Scientific in 0.1% TTBS). After blocking, the membranes were incubated overnight, under agitation, with the following primary antibodies: β-actin (Millipore Cat# MAB1501, RRID:AB_2223041) (1:1000), P-FAK (Tyr^397^) (Millipore Cat# ABT135, RRID:AB_10947863) (1:1000), and integrin β1 (Millipore Cat# MAB2000, RRID:AB_94503) (1:500) from Millipore (Burlington, MA, USA); α-tubulin (Sigma-Aldrich Cat# T9026, RRID:AB_477593) (1:1000) and vimentin (Sigma-Aldrich Cat# V6389, RRID:AB_609914) (1:1000) from Sigma-Aldrich (St. Louis, MO, USA); alkaline phosphatase (Thermo Fisher Scientific Cat# 702454, RRID:AB_2722857) (1:1000) and collagen type I (Thermo Fisher Scientific Cat# PA5-95137, RRID:AB_2806942) (1:500) from INVITROGEN (Waltham, MA, USA); osteopontin (Santa Cruz Biotechnology Cat# sc-21742, RRID:AB_2194997) (1:500), integrin α2 (Santa Cruz Biotechnology Cat# sc-74466, RRID:AB_1124939) (1:500), integrin α5 (Santa Cruz Biotechnology Cat# sc-59762, RRID:AB_1123667) (1:500), α-smooth muscle actin (Santa Cruz Biotechnology Cat# sc-32251, RRID:AB_262054) (1:1000), PPAR-γ (Santa Cruz Biotechnology Cat# sc-7273, RRID:AB_628115) (1:1000), and TGF-β1/2/3 (Santa Cruz Biotechnology Cat# sc-7892, RRID:AB_677514) (1:500) from Santa Cruz Biotechnology (Dallas, TX, USA); FAK (Abcam Cat# ab40794, RRID:AB_732300) (1:1000) and CEBP-α (Abcam Cat# ab40764, RRID:AB_2077888) (1:1000) from Abcam (Cambridge, UK); P-β-catenin (Ser33/37/Thr41) (Cell Signaling Technology Cat# 9562, RRID:AB_331149) (1:1000) from Cell Signalling (Danvers, MA, USA); and CD90 (Proteintech Cat# 66766-1-Ig, RRID:AB_2882112) (1:1000) from Proteintech (Rosemont, IL, USA). After that, the membranes were washed and incubated with a biotinylated secondary antibody for 60 min, followed by further streptavidin incubation (Cat#434323; Thermo Fisher Scientific, Waltham, MA, USA). Finally, the proteins of interest were detected using the ChemiDoc Imaging System (BIO-RAD, CA, USA). The quantification of bands was performed using the ImageLab software for PC version 6.1 from BIO-RAD.

### 2.8. Immunofluorescence

Immunofluorescence was performed according to a protocol described previously [22]. SAOS-2 cells were seeded on glass coverslips previously covered with 1% gelatin, at a density of 3 × 10^4^ cells per well (1.58 × 10^4^ cells/cm^2^) in 24-well plates. After 3 days, the cells were treated with 20% LATS or OATS for an additional 3 days. After stimulation, the cells were washed once with 1× PBS at 37 °C, fixed in a 4% paraformaldehyde/4% sucrose/1× PBS solution for 30 min, washed again with 1× PBS, permeabilized with a 0.2% Triton-X solution in 1× PBS, blocked with 5% BSA in 1× PBS for 45 min, and incubated overnight with the respective primary antibodies, diluted in 1% BSA in 1× PBS at a concentration of 1:400 at 4 °C. The next day, the coverslips were washed 3 times with 1× PBS; incubated for 60 min at 4 °C with the biotinylated secondary antibody anti-rabbit (Cytiva, Marlborough, MA, USA, Cat# RPN1001-2ML, RRID:AB_1062579) or anti-mouse (Cytiva Cat# RPN1004-2ML, RRID:AB_1062582) (diluted in 1% BSA in 1× PBS at a concentration of 1:400); washed 3 times with 1× PBS; and incubated with the fluorochrome ALEXA 555 (diluted in 1% BSA in 1× PBS at a concentration of 1:400) for 60 min. After this last incubation, the coverslips were washed with 1× PBS, counterstained, and mounted for visualization with DAPI Prolong (Cat#P36935), Invitrogen (Waltham, MA, USA), under a microscope (Model Olympus IX71 Olympus, Tokyo, Japan) equipped for epifluorescence at 600× to 1000× magnification. The mesenchymal markers CD90, vimentin, and α-smooth muscle actin and the adipogenic marker perilipin (Thermo Fisher Scientific Cat# PA5-55046, RRID:AB_2645393)—Invitrogen (Waltham, MA, USA)—were observed.

### 2.9. Adipogenic Transdifferentiation Assay—Oil Red Staining

**Scheme 2:**
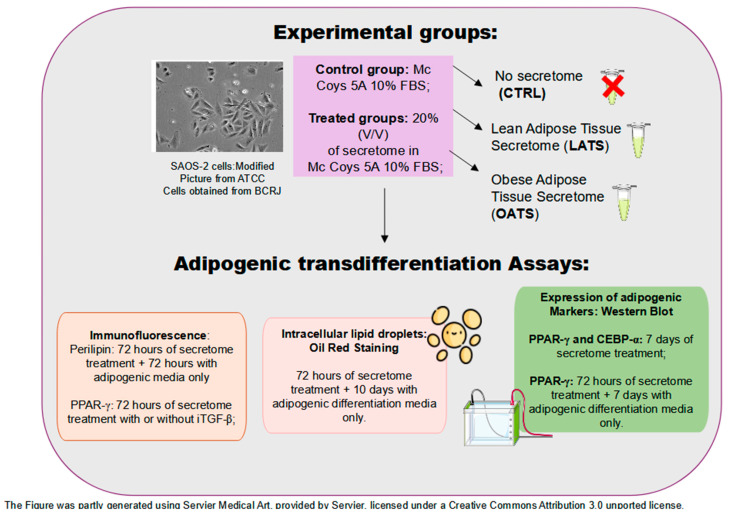
Adipogenic transdifferentiation experimental design. SAOS-2 cells were pretreated with the lean adipose tissue secretome (LATS), the obese adipose tissue secretome (OATS), or no secretome (CTRL) for 72 h. After that, the cells were incubated in adipogenic differentiation culture media. Immunofluorescence, oil red staining, and a Western blot were performed at specific time points.

SAOS-2 cells were seeded at a density of 1.5 × 10^4^ cells per well in a 96-well plate (4.7 × 10^4^ cells/cm^2^). After 24 h, the cells were pretreated with an osteogenic differentiation medium supplemented with 20% LATS or 20% OATS for 3 days. Subsequently, the culture medium was removed entirely, and the adipogenic differentiation medium was added to all the experimental groups equally. The adipogenic differentiation medium was McCoy’s 5A with 10% FBS supplemented with 10 μM insulin, 1 μM dexamethasone, and 0.5 mM isobutylmethylxanthine. The pre-treated cells were cultured in an adipogenic medium for 10 days, with medium changes every 3–4 days. For the visualization of intracellular lipid droplets, oil red staining was performed as previously described [23]. Briefly, the cells were washed once with 1× PBS at 37 °C, fixed with 4% paraformaldehyde for 10 min, washed once with 1× PBS, and incubated with an oil red-O (Cat#O0625) (Sigma-Aldrich, St. Louis, MO, USA) working solution for 15 min at room temperature. The staining was removed and the cells were washed with distilled water 3 times and observed under a microscope at 600× magnification (Model Olympus IX71 Olympus, Tokyo, Japan). The adipogenic transdifferentiation assays are illustrated in Scheme 2.

### 2.10. Quantification of Adipokines

The panel of adipokines present in the secretomes released by the subcutaneous AT of lean individuals and individuals with obesity was performed according to the manufacturer’s instructions for use. To measure IL-6 (detection limit: 13 pg/mL) and TNF-α (detection limit: 0.5 pg/mL), the Human Metabolic Hormone Bead Panel kit (Millipore—Cat. #HMHEMAG-34K) was used. To measure IL-1β (detection limit: 0.8 pg/mL) and MCP-1 (detection limit: 1.9 pg/mL), the Human Cytokine/Chemokine Magnetic Bead Panel kit (Millipore—Cat. #HCYTOMAG-60K) was used. The TGF-β 1, 2, 3 Magnetic Bead Kit (Millipore—Cat. #TGFBMAG-64K-03) was used to measure TGFβ1, TGFβ2, and TGFβ3 (detection limits: 6.0, 6.6, and 2.2 pg/mL, respectively).

### 2.11. Gas Chromatography–Mass Spectrometry (GC–MS)

The content of long-chain fatty acids in the secretomes was analyzed in a volume corresponding to 500 μg of proteins using GC–MS, as previously described [24]. Lipid samples were dissolved in 1 mL of toluene and 2 mL of 1% sulfuric acid in methanol. After 24 h in a stoppered tube at 50 °C, 1 mL of 5% NaCl was added, and the required esters were extracted (2X) with 2 mL of hexane and then removed in a stream of nitrogen. Dried fatty acid methyl esters (FAMEs) were suspended in 100 μL of heptane. The GC–MS analyses were carried out on a Shimadzu, (Kyoto, Japan) GCMS-QP2010 Plus system, using an HP Ultra 2 (5% phenyl-methylpolysiloxane) and Agilent (Santa Clara, CA, USA) (25 m × 0.20 mm × 0.33 μm). The splitless injector was set to 250 °C. The column temperature was programmed to increase from 40 to 160 °C at 30 °C/min, 160 to 233 °C at 1 °C/min, and 233 to 300 °C at 3 °C/min, and was held at 300 °C for 10 min. We used helium as the carrier gas with a linear velocity of 36.0 cm/s. Then, 2 mL of the sample was injected into the chromatograph. Electron ionization (EI-70 eV) and a quadrupole mass analyzer performed the analysis in scans from 40 to 440 amu. The interface was set to 240 °C, and the ion source was set to 240 °C. The components were identified by comparing their mass spectra with those of the library NIST05 contained in the computer of the mass spectrometer. We used retention indices to confirm the identity of the peaks in the chromatogram with the Supelco 37 Component FAME Mix (Sigma-Aldrich). The FFAs were quantified by determining the peak–area ratios with the internal standards 9:0 and 19:0.

### 2.12. Total Free Fatty Acid Dosage

The total free fatty acid quantification was performed using a Free Fatty Acid Quantitation Kit (Sigma-Aldrich—Cat. #MAK044) on the OATS and LATS samples in accordance with the manufacturer’s instructions.

### 2.13. Statistical Analysis

The data distribution regarding the normality in the experiments using secretomes from both groups (obese and lean) was checked (Shapiro–Wilk test, QQ plot).

The graphics and statistical tests were created/performed using the GraphPad Prism software, version 7.0 (GraphPad Prism, RRID:SCR_002798) (GraphPad Software, La Jolla, CA, USA). The data were available as means and the standard deviations of the means. A one-way ANOVA test was performed, followed by the Bonferroni post-test for multiple comparisons and Student’s *t*-test for comparisons between two groups. In all the analyses, *p* < 0.05 was considered statistically significant. The outliers identified through a Graphpad Prism (GraphPad Prism, RRID:SCR_002798) outlier calculation software analysis were removed from the statistical analysis.

A parametric independent *t*-test was selected for the adipokine data analysis. Statistically significant results with a biological relevance were respectively considered for two-tailed *p*-values < 0.05. The results were evaluated using the mean ± standard error. The SPSS 20 software (IBM Inc., Chicago, IL, USA) and the R language for Windows (version 4.2.2, in the Rstudio environment and Rmarkdown format) were selected to carry out the analyses.

## 3. Results

### 3.1. Secretome of Obese Adipose Tissue Increases Osteoblast Proliferation and Changes Cell Morphology

**Figure 1:**
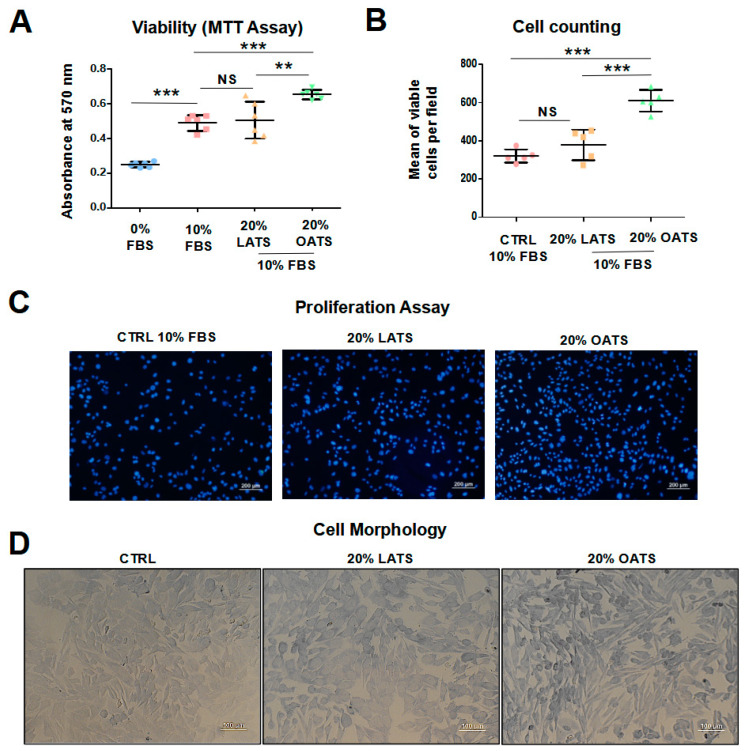
Secretome of obese adipose tissue enhanced osteoblast proliferation and changed cell morphology. (**A**–**C**) SAOS-2 cells were incubated in medium without secretome (0% FBS), medium with 10% FBS without secretome (CTRL), or treated with lean adipose tissue secretome (20% LATS) or obese adipose tissue secretome (20% OATS) for 48 h. (**A**) OATS increased cell viability, as assessed by MTT assay. (**B**,**C**) OATS increased cell proliferation, as assessed by DAPI-stained cell counting after 48 h. (**D**) OATS induced alterations in SAOS-2 cell morphology, which assumed elongated spindle shape in 72 h. Images are representative of six independent experiments. Data are expressed as mean ± SD of 5–6 subjects per group. One-way ANOVA–Bonferroni post-test was performed; ** *p* < 0.01, *** *p* < 0.001, NS, Not significant.

To evaluate the interference of the adipose tissue secretome on the proliferation of osteoblasts, SAOS-2 cells were treated with the secretome derived from lean (LATS) or obese (OATS) adipose tissue for 48 h. The cell viability was determined by an MTT assay, and proliferation was evaluated by cell counting in DAPI-stained cell nuclei. Figure 1 shows that the OATS treatment increased the cell viability (Figure 1A) and proliferation (Figure 1B,C) when compared with the control group (10% FBS) or with the LATS-treated cells. To discard the effect of FBS on cell viability and proliferation, assays were performed in a 0% FBS medium, and similar results were obtained in SAOS-2 cells stimulated with the OATS (Supplementary Figure S1).

In the osteogenic differentiation stages, more elongated or spindle-shaped cells are associated with a low commitment to maturation. To assess changes in cell morphology, SAOS-2 cells were either left untreated (CTRL) or were treated with the OATS or LATS. Following the treatment, the cells were fixed and stained with methylene blue for visualization under optical microscopy. Figure 1D illustrates that, after three days of the OATS treatment, the SAOS-2 cells adopted a spindle-shaped morphology indicative of cellular dedifferentiation. In contrast, the cells in the CTRL group or those treated with the LATS retained their characteristic cuboidal shape (Figure 1D).

### 3.2. Secretome of Obese Adipose Tissue Reduces Early and Late Osteoblast Differentiation Markers and Impairs Extracellular Matrix Mineralization

**Figure 2:**
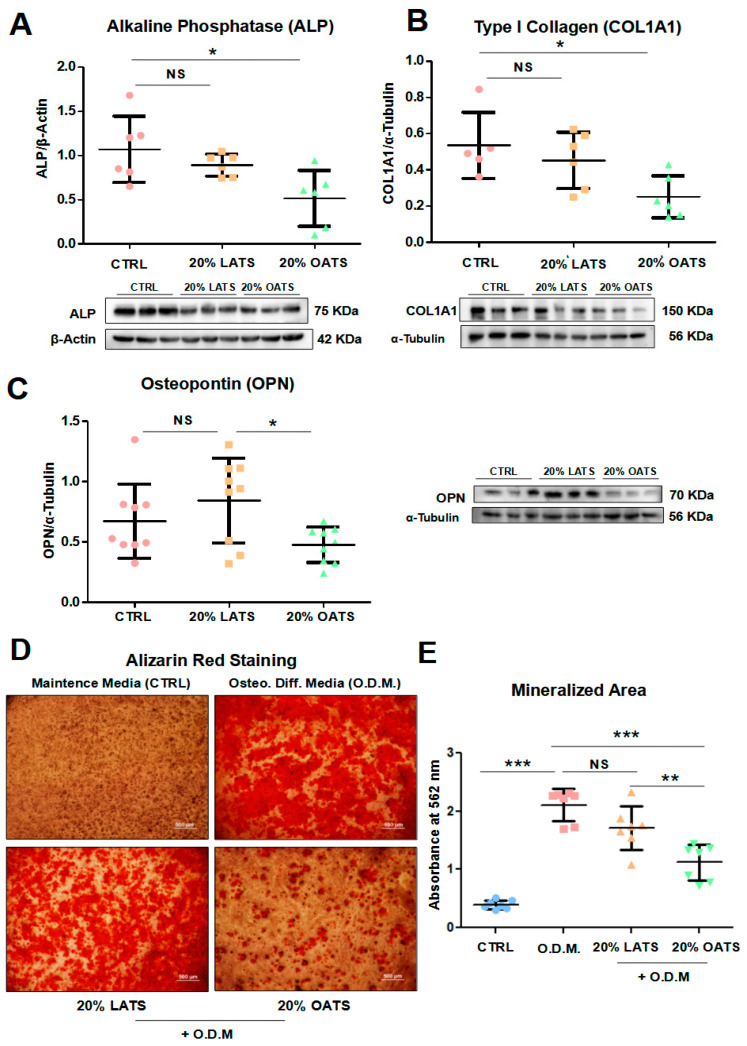
Secretome of obese adipose tissue reduced early and late osteoblast differentiation markers and impaired extracellular matrix mineralization. SAOS-2 cells incubated in osteogenic differentiation medium (O.D.M) were treated with LATS, OATS, or 10% FBS (CTRL) for 7 days. (**A**–**C**) Protein expression of early and late osteoblast differentiation markers was analyzed. Alkaline phosphatase (ALP) (**A**); collagen type I (COL1A1) (**B**); and osteopontin (OPN) (**C**). (**D**,**E**) Treatment with OATS (20%) inhibited extracellular matrix mineralization, as assessed by alizarin red staining (**D**) and quantified by D.O. (**E**) in cells cultured in O.D.M. Data are expressed as mean ± SD for groups of 7–9 subjects. One-way ANOVA–Bonferroni post-test was used; * *p* < 0.05, ** *p* < 0.01, *** *p* < 0.001, NS, Not significant.

To assess the influence of the AT secretomes on osteoblast differentiation, SAOS-2 cells were treated with an osteogenic differentiation medium (O.D.M.), with or without 20% LATS or OATS. The stages of osteogenic differentiation were evaluated through the protein expression of early and late markers, using alkaline phosphatase (ALP) and collagen type I (COL1A1) as initial markers and osteopontin (OPN) as a late marker [25]. The cells treated with a differentiation medium containing the OATS, but not with the medium containing the LATS, presented a reduced expression of ALP (Figure 2A) and COL1A1 (Figure 2B) when compared to control cells incubated only with the differentiation medium (O.D.M.). Furthermore, our results demonstrated that cells treated with the OATS presented a reduced expression of OPN (Figure 2C) when compared to those treated with the LATS. Treatment with the LATS did not modify the expression of any markers when compared to cells treated with the differentiation medium alone.

Once differentiated, mature osteoblasts acquire the ability to accumulate and mineralize the organic extracellular matrix [6]. To evaluate the impact of the adipose tissue secretomes on cell mineralization under healthy and obese conditions, SAOS-2 cells were incubated for 10 days with an osteogenic medium and stained with alizarin red. The effect of the OATS and LATS on mineralization was visualized using microscopy (Figure 2D), and the alizarin red staining was quantified (Figure 2E). The results showed that the treatment with the OATS inhibited bone extracellular matrix mineralization compared to the O.D.M. or LATS treatment.

### 3.3. Secretome of Obese Adipose Tissue Enhances Mesenchymal Marker Expression of Osteoblasts

**Figure 3:**
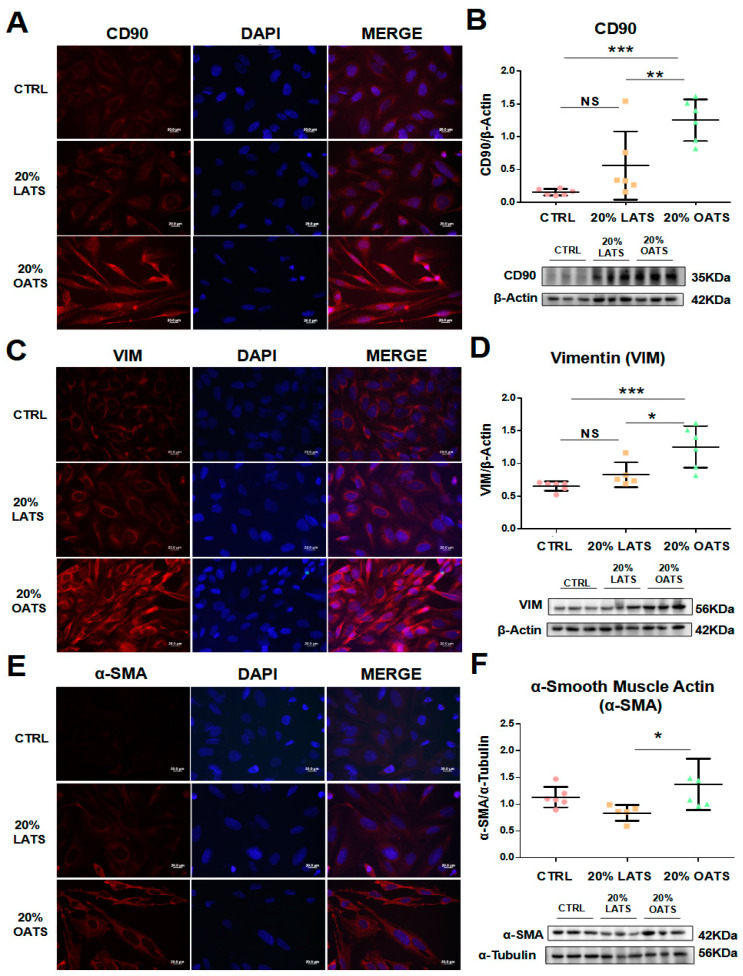
Secretome of obese adipose tissue enhanced mesenchymal marker expression of osteoblasts. SAOS-2 cells incubated in osteogenic differentiation medium (O.D.M) were treated with LATS, OATS, or 10% FBS (CTRL) for 3 days and expression of mesenchymal markers was evaluated. (**A**,**C**,**E**) Increased immunofluorescence using Alexa Fluor 555 was observed in cells treated with 20% OATS for CD90 (**A**), vimentin (**C**), and α-smooth muscle actin (α-SMA) (**E**). DAPI-stained nuclei are shown in blue. Representative images of 4 independent experiments (600×). (**B**,**D**,**F**) Protein expression of CD90 (**B**), vimentin (**D**), and α-SMA was increased by OATS (**F**). Data are expressed as mean ± SD per group of 6 subjects. One-way ANOVA–Bonferroni post-test was used; * *p* < 0.05, ** *p* < 0.01, *** *p* < 0.001, NS, Not significant.

To investigate a possible phenotype regression of osteoblasts treated with the OATS, we analyzed the expression of CD90, vimentin (VIM), and α-smooth muscle actin (α-SMA), three known mesenchymal stem cell (MSC) markers [26,27,28]. The immunofluorescence images of cells treated for 3 days with the OATS showed that the SAOS-2 cells presented a spindle-shaped morphology accompanied by increasing fluorescence for CD90 (Figure 3A), VIM (Figure 3C), and α-SMA (Figure 3E). Treatment with the LATS did not modify the cuboid morphology, maintaining a low expression of those osteoprogenitor markers, as with the control cells (CTRL). A Western blot analysis confirmed the commitment of SAOS-2 cells towards a mesenchymal phenotype, revealing the increased protein expression of CD90 (Figure 3B), VIM (Figure 3D), and α-SMA (Figure 3F) when the cells were treated with the OATS, in contrast to the CTRL or the LATS treatment.

### 3.4. Secretome of Obese Adipose Tissue Affects Integrin β1 Expression, FAK, and β-Catenin Signaling Pathway

**Figure 4:**
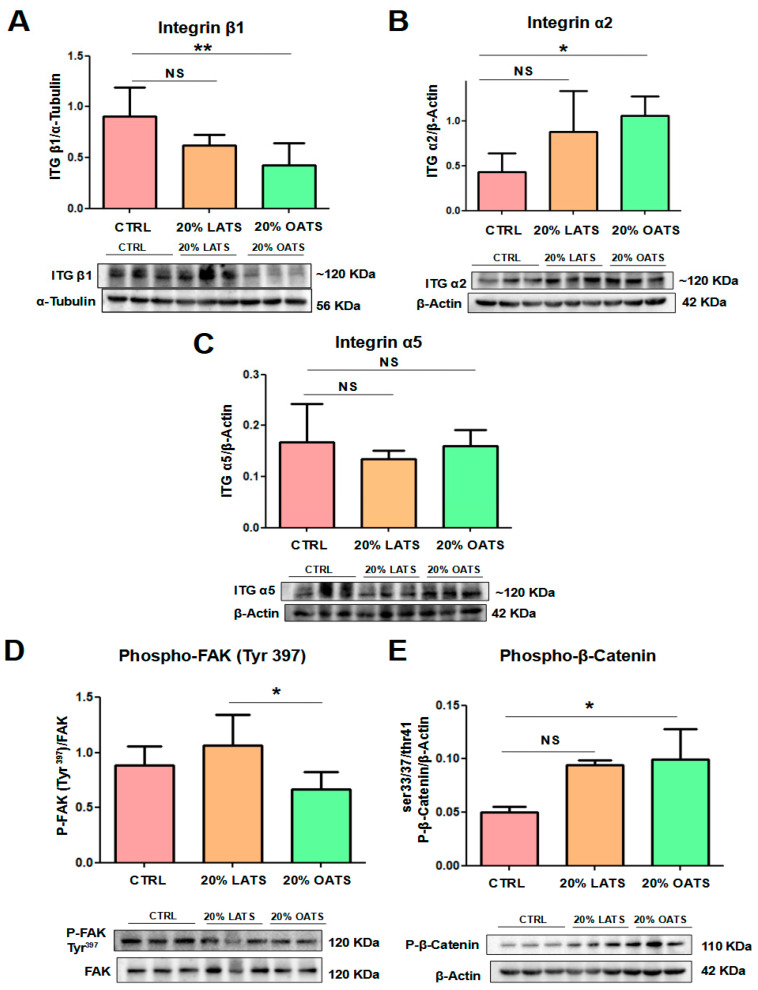
Obese adipose tissue secretome impacts integrin β1 expression and FAK-β-catenin signaling pathway. (**A**–**C**) SAOS-2 cells were treated with LATS, OATS, or 10% FBS (CTRL) for 7 days. (**A**–**C**) Expression of integrin β1 (**A**), integrin α2 (**B**), and integrin α5 (**C**). (**D**,**E**) Phosphorylation of focal adhesion kinase (^Tyr397^pFAK) (**D**) and β-catenin (^Ser33−37/Thr41^pβCat) (**E**). Data are expressed as mean ± SD per groups of 5–6 subjects. One-way ANOVA–Bonferroni post-test was used; * *p* < 0.05; ** *p* < 0.01, NS, Not significant.

Integrins have a fundamental role in osteoblast maturation through their interaction with the extracellular matrix proteins. α2β1 integrin is the main adhesion receptor of type I collagen, and α5β1 binds to OPN, bone sialoprotein, and fibronectin [29]. Figure 4 shows that the stimulation of SAOS-2 cells with the OATS significantly reduced the expression of β1 integrin (Figure 4A) while increasing α2 integrin (Figure 4B), and it did not interfere with the expression of the α5 subunit (Figure 4C) compared to the control group. The cells treated with the LATS did not show significant alterations in integrin subunit expression compared to the control group.

Integrin signaling mediates proliferation, survival, and osteogenic differentiation [29]. In osteoblasts, integrin activation triggers FAK phosphorylation, activating the downstream PI3K-AKT pathway and allowing β-catenin translocation to the nucleus to mediate osteogenic gene expression [30]. The treatment of SAOS-2 cells with the OATS, but not the LATS, inhibited FAK phosphorylation at the Tyr^397^ residue (Figure 4D) and increased β-catenin phosphorylation (Figure 4E).

### 3.5. Secretome of Obese Adipose Tissue Enhances an Adipogenic Profile in Osteoblasts

**Figure 5:**
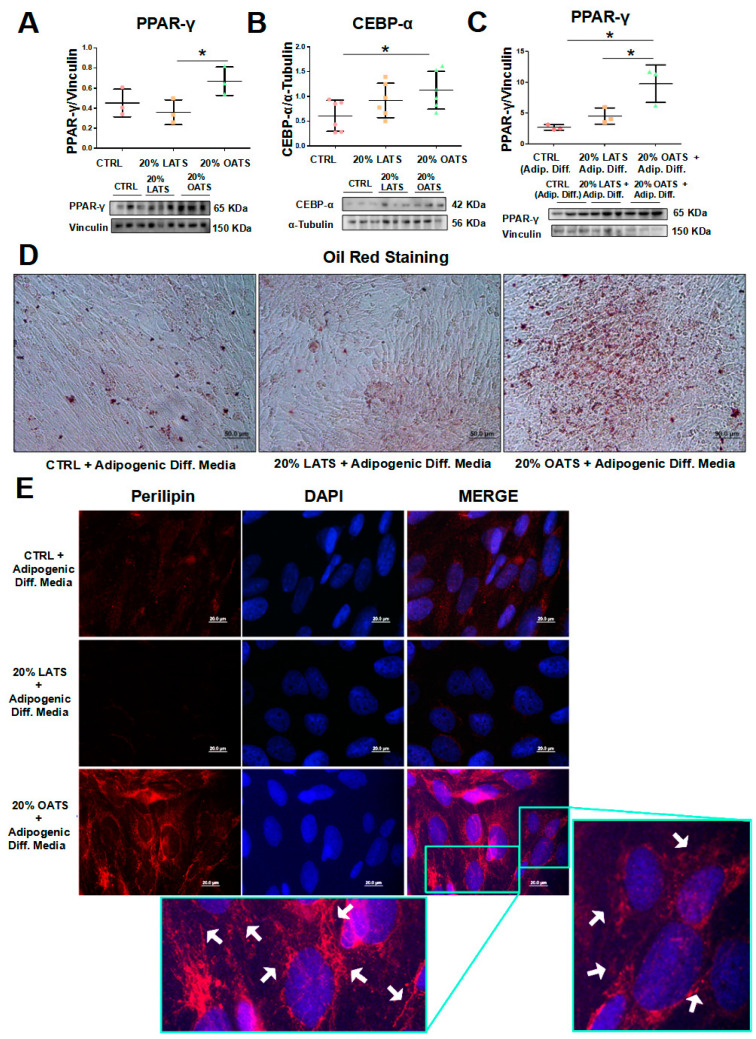
Obese adipose tissue secretome stimulates adipogenic marker expression and lipid accumulation in osteobasts. (**A**,**B**) SAOS-2 cells were treated with LATS, OATS, or 10% FBS (CTRL) for 7 days. PPAR-γ (**A**) and CEBP-α (**B**) protein expression was analyzed. Data are expressed as mean ± SD of 4 (**A**) and 6 (**B**) subjects. (**C**–**E**) Cells were treated with LATS, OATS, or 10% FBS (CTRL) for 72 h, and further cultured in adipogenic differentiation medium from 7 to 10 days. (**C**) PPAR-γ protein expression (after 7 days). Data are mean ± SD of 5 subjects per groups. (**D**) OATS (20%) treatment increased lipid droplet accumulation in SAOS-2 cells, as indicated by enhanced oil red-O staining after 10 days of incubation in adipogenic differentiation medium. Representative images (400×), with four subjects per group. (**E**): OATS (20%) also increased immunofluorescence staining for perilipin (Alexa Fluor 555) after 3 days of incubation in adipogenic differentiation medium; representative images (1000×) of four subjects per group. INSERT: highlighted perilipin (in red) surrounding intracellular lipid droplets, as indicated by white arrows. One-way ANOVA followed by the Bonferroni post-test (* *p* < 0.05) was used for the statistical analysis.

So far, the results indicate that the OATS might lead osteoblasts to dedifferentiation towards an osteoprogenitor phenotype. Next, we investigated whether dedifferentiation can result in SAOS-2 cells moving towards an adipogenic profile. Accordingly, we observed the increased expression of PPAR-γ (Figure 5A) and CEBP-α (Figure 5B) in cells treated with the OATS for 7 days. Treatment with the LATS did not modify the expression of adipogenic transcription factors compared to the control. Pre-treatment with the OATS for 3 days and a subsequent 7 days of the adipogenic differentiation protocol also increased the PPAR-γ expression (Figure 5C). To evaluate whether the pre-treatment with the OATS would favor the osteoblasts to assume an adipocyte-like phenotype, the cells were stained with red oil, and the intracellular lipid storage was analyzed by microscopy. The SAOS cells pre-treated with the OATS or LATS and cultured for a subsequent 10 days in an adipogenic differentiation medium showed increased intracellular lipid droplet accumulation, with a group of cells assuming an adipocyte-like profile in the OATS group (Figure 5D). This behavior was not observed in the cells pre-treated with the LATS. Supporting these results, the immunofluorescence images showed that the cells pre-treated with the OATS presented an enhanced expression of perilipin (Figure 5E). The white arrows indicate the presence of circular structures formed by perilipin surrounding lipid droplets on osteoblasts pre-treated with the OATS (Figure 5E).

### 3.6. Adipokine and Free Fatty Acid Secretion by Subcutaneous Adipose Tissue In Vitro

**Figure 6:**
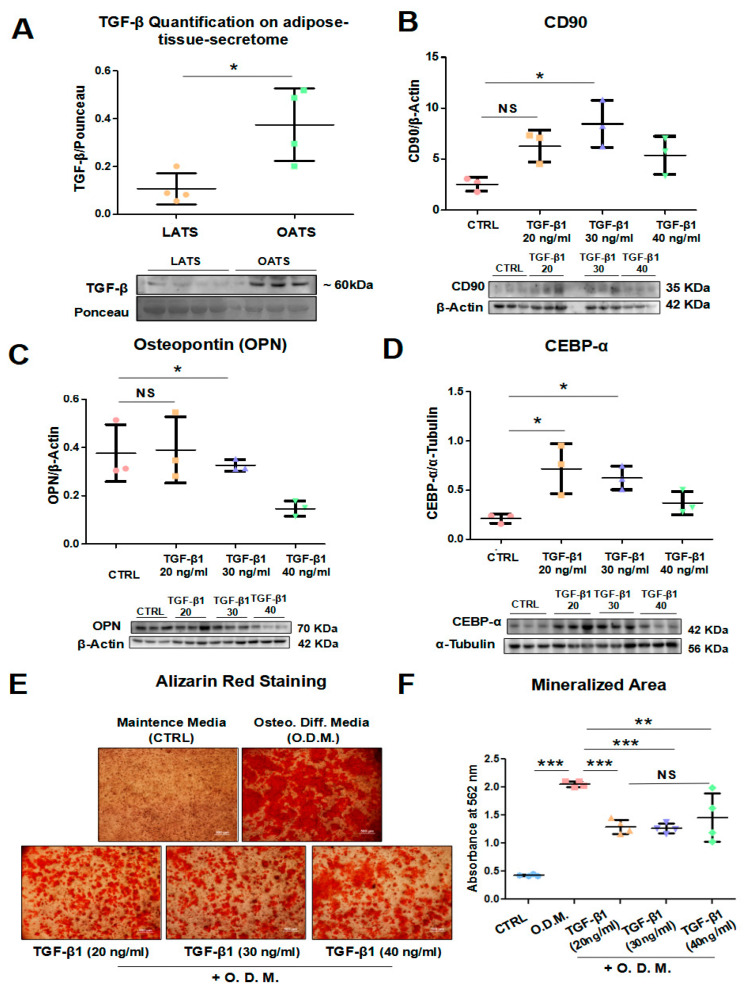
TGF-β1 stimulation induced effects similar to OATS on SAOS-2 cells. (**A**) Quantification of TGF-β concentration in OATS and LATS. (**B**–**D**) Effect of TGF-β1 on mesenchymal and osteogenic markers: CD90 (**B**), osteopontin (OPN) (**C**), and CEBP-α (**D**). Data are expressed as mean ± SD per group of at least 4 subjects. (**E**,**F**) TGF-β1 inhibited ECM mineralization: SAOS-2 cells were incubated in O.D.M. and treated for 10 days with rTGF-β1 (20, 30, or 40 ng/mL) or 10% FBS (CTRL). rTGF-β1 diminished ECM deposition, as observed through lower alizarin red staining (**E**) and further quantified by D.O. (**F**). Representative images (40×). Data are expressed as mean ± SD per group of at least 4 subjects. One-way ANOVA–Bonferroni post-test was performed; * *p* < 0.05, ** *p* < 0.01, *** *p* < 0.001, NS, Not significant.

The examination of adipokines secreted by subcutaneous adipose tissues revealed that, despite some variability among individual samples, the content of most adipokines did not exhibit significant differences in the secretome between obese and lean adipose tissues (Supplementary Figure S2). However, the OATS displayed elevated levels of TGF-β1 (1.67-fold, *p* = 0.006) compared to the LATS, with no significant differences in the MCP1, IL1β, TNFα, leptin, IL6, TGF-β2, or TGF-β3 concentrations (Supplementary Figure S2B). An increase in TGF-β1 in the OATS was further confirmed by a Western blot (Figure 6A). Notably, a moderate positive correlation between TGFβ1 and BMI was observed (r = 0.602; *p* = 0.030), whereas no correlation was found between other adipokine levels and the patient’s BMI (Supplementary Figure S2C). Notably, the adipokine profiles of the male participants with obesity did not differ significantly from those of the female participants with obesity. This consistency was observed across the various results in the study, despite individual differences.

Additionally, we assessed both the total content and the profile of the unsaturated free fatty acids released by lean and obese subcutaneous adipose tissue. A higher total fatty acid content was identified in the OATS compared to the LATS (Supplementary Figure S2E). Nevertheless, no distinctions were observed between the two tissues regarding the individual content of unsaturated free fatty acids released in the secretomes derived from the subcutaneous adipose tissue of lean patients and patients with obesity (Supplementary Figure S2D).

### 3.7. TGF-β1 Contributes to OATS-Induced Dedifferentiation of Osteoblasts

Increased circulating levels and the augmented adipose tissue expression of TGF-β have been reported during human obesity [31]. Supporting these data, we detected higher levels of TGF-β1 in the secretome of the subcutaneous adipose tissue of obese patients, compared to the LATS (Figure 6A). TGF-β1 is a known critical regulator of osteogenic differentiation [32] and a putative key molecule in the dedifferentiation effect of the OATS on osteoblasts.

To validate the hypothesis that the effect of the OATS on osteoblast dedifferentiation was TGF-β1 dependent, SAOS-2 cells were stimulated with different concentrations of hrTGF-β1 (20, 30, and 40 ng/mL). The treatment with TGF-β1 maintained cell viability and increased cell proliferation at all the concentrations tested (Supplementary Figure S3A,C,D). These effects were accompanied by pronounced morphological changes, shifting cells towards a fibroblastic morphology, which was more evident at higher concentrations (Supplementary Figure S3B). The TGF-β1 treatment also reduced the expression of the late differentiation marker OPN (Figure 6C), decreased FAK phosphorylation at 40 ng/mL (Supplemental Figure S3F), and increased β-catenin phosphorylation (Supplemental Figure S3E), while enhancing the expression of the mesenchymal marker CD90 (Figure 6B) and the adipogenic marker CEBP-α (Figure 6D). Additionally, TGF-β1 inhibited extracellular matrix calcification by SAOS-2 cells cultured for 10 days in an osteogenic differentiation medium when compared to the O.D.M. group (Figure 6E,F). Together, the results showed that TGF-β1 can induce similar effects to the OATS on SAOS-2 cells.

**Figure 7:**
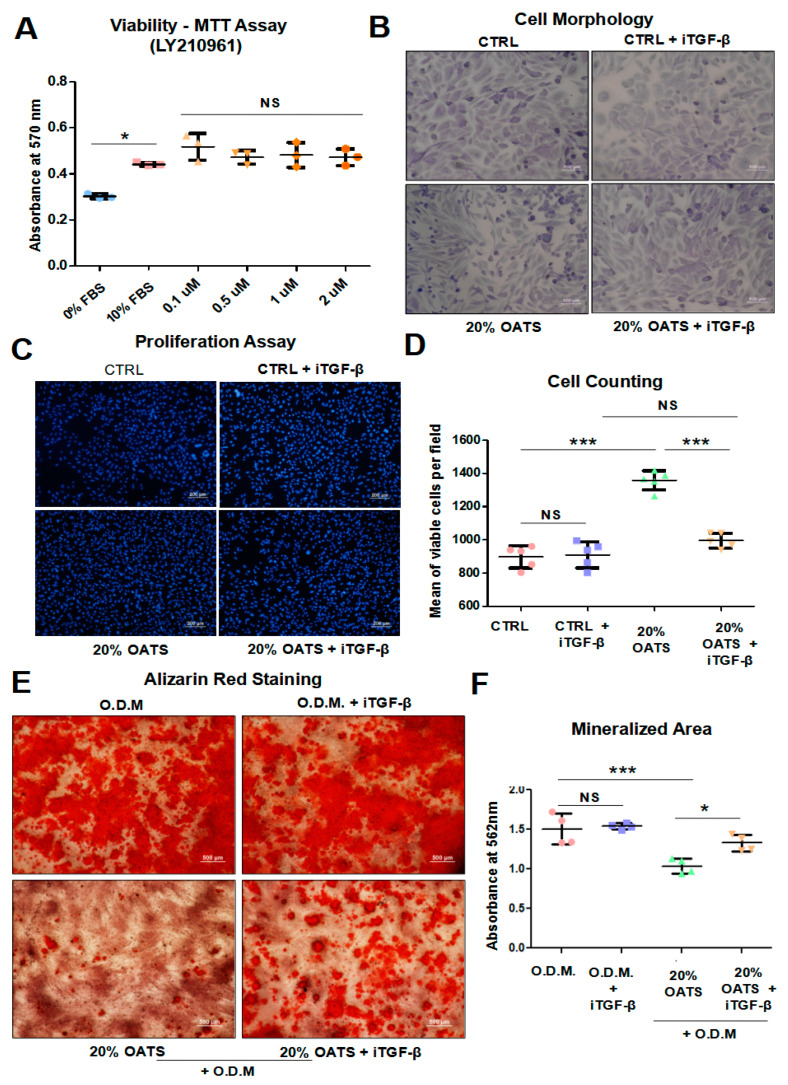
TGF-β blockade attenuated effects of OATS on osteoblasts. (**A**) SAOS-2 cell viability (MTT assay) in presence of LY210961 (iTGF-β: 0.1–2.0 μM) for 48 h. (**B**–**E**) iTGF-β (1 μM) treatment inhibited OATS (20%)-induced changes in cell morphology (after 3 days), preventing osteoblasts from assuming mesenchymal-type shape (**B**) and inhibiting osteoblast proliferation, as assessed by DAPI-stained cell counting after 48 h (**C**,**D**). Treatment with iTGF-β impaired OATS inhibitory effect on ECM mineralization, as observed through increasing alizarin red staining in cells treated with 20% OATS+ iTGF-β (1 µM) (**E**,**F**)**.** Representative images (40×). Data are expressed as mean ± SD for groups of 4–5 subjects. One-way ANOVA followed by Bonferroni post-test was used (* *p* < 0.05, *** *p* < 0.001, NS, Not significant).

**Figure 8:**
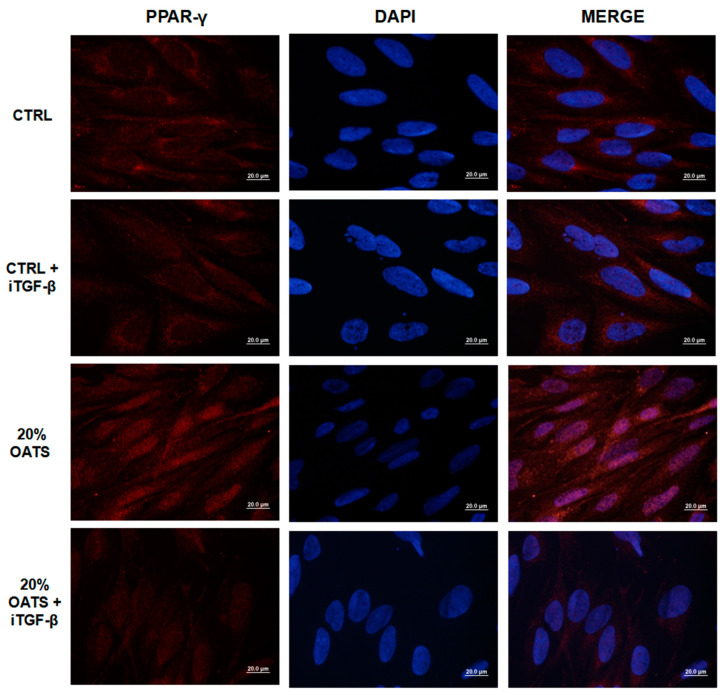
Blockade of TGF-β inhibited OATS-induced PPAR-γ expression and nuclear translocation. Treatment with 20% OATS for 72 h increased PPAR-γ immunofluorescence (ALEXA 555, red) in nuclei of DAPI-stained SAOS-2 cells. Incubation with iTGF-β (1 µM) suppressed OATS-induced increase in PPAR-γ expression (20% OATS+ iTGF-β). Representative images (1000× magnification) were obtained from at least three samples per group.

To demonstrate that the increasing levels of TGF-β1 in the secretome of obese adipose tissue are involved in the dedifferentiation effects of the OATS on osteoblasts, osteoblasts were treated with the OATS in the presence of the TGF-β receptor antagonist (iTGF-β), LY210961. An MTT assay demonstrated that, at concentrations as high as 2 μM, the antagonist did not compromise cell viability (Figure 7A). Figure 7 shows that iTGF-β, used at 1 μM, impaired the morphological alterations induced by the OATS on SAOS-2 cells (Figure 7B), reversed the increased proliferative capacity (Figure 7C,D), and partially restored the ability of osteoblasts to promote extracellular matrix calcification (Figure 7E,F). Additionally, the blockage of the TGF-β receptor impaired the OATS-mediated PPAR-γ expression and, consequently, its nuclear translocation, as detected by immunofluorescence microscopy (Figure 8). The results indicate that the TGF-β present in the OATS could be a key driver in the dedifferentiation of osteoblasts towards an adipogenic profile.

## 4. Discussion

The relationship between obesity and bone health is not entirely straightforward. While an increased mechanical load may lead to greater bone density, the negative impacts of inflammation, hormonal imbalances, and vitamin deficiencies can offset these benefits. In our work, we evaluated how the released factors from the human subcutaneous adipose tissue of individuals with obesity can directly affect the stages of differentiation of human osteoblasts. We provided novel and consistent data showing that the secretome of obese subcutaneous adipose tissue, enriched in TGF-β1, has a substantial impact on osteoblast maturation and differentiation.

The results show that, in contrast with the secretome derived from the subcutaneous adipose tissue of lean patients (LATS), the obese adipose microenvironment (OATS) increased the viability and proliferation of osteoblasts, which exhibited a spindle-shaped morphology, a feature associated with a lower commitment to maturation. The influence of the OATS on osteoblast differentiation was evident through the reduced expression of early and late differentiation markers, such as alkaline phosphatase (ALP), type I collagen (COL1A1), and osteopontin (OPN).

The influence of adipocytes on osteoblast differentiation, in vitro, was reported earlier using co-cultures of pre-adipocyte (3T3-L1) and pre-osteoblast (MC3T3-E1) cell lines [33]. However, to the best of our knowledge, our study showed for the first time the direct effect of human subcutaneous adipose tissue secretions on cultures of mature human osteoblasts, interfering with their differentiation in obesity. In line with the data, studies in vivo have reported significant trabecular and cortical bone loss and a decrease in osteoblast number on bone surfaces induced by obesity in rodents [34,35,36].

During the early stages of the bone-remodeling process, an increasing number of αSMA+ osteoprogenitor cells is found, decreasing during osteoblast maturation, as the cells substantially increase the expression of osteocalcin and osteoprotegerin, late markers of osteoblastic differentiation [37]. Interestingly, we observed that, besides the alterations in cell morphology, the proliferative behavior, and the loss of osteogenic markers, the OATS appeared to induce a phenotype regression in osteoblasts, leading to the increased expression of mesenchymal stem cell markers, such as CD90, vimentin, and α-smooth muscle actin. These results, added to the fact that the OATS also inhibited extracellular matrix mineralization, a crucial feature of mature osteoblasts, strongly suggest that osteoblasts in contact with the secretome of obese adipose tissue undergo a dedifferentiation process.

Supporting that premise, our research revealed that the secretome from obese adipose tissue was found to have a notable impact on key molecular pathways, known to be involved in osteoblast maturation and subsequent osteogenic differentiation [29]. Specifically, the OATS significantly reduced the expression of β1 integrin, inhibited FAK phosphorylation, and increased β-catenin phosphorylation. The conditional deletion of β1 integrin was shown earlier to impact osteogenesis, leading to bone loss in young mice [38]. Besides that, the OATS increased the α2 subunit expression, which is usually upregulated in undifferentiated stages of osteoblasts [39].

Integrin-mediated adhesions and their interplay with the Wnt/β-catenin signaling pathway play pivotal roles in the regulation of osteogenic differentiation and mechanotransduction in osteoblasts. This intricate process involves a complex signaling cascade, including the activation of FAK and PI3K/Akt pathways and the inhibition of GSK3β, thus preventing the phosphorylation of β-catenin and allowing its translocation to the nucleus to mediate osteogenic gene expression [29]. The phosphorylation of β-catenin reduces its activation by favoring its degradation, leading to inhibitory effects on osteoblastogenesis [30].

The Wnt/β-catenin signaling pathway is known to be highly activated in mesenchymal precursor cells, particularly those committed to the osteoblast lineage while inhibiting their differentiation into adipocytes [40]. The suppression of Wnt/β-catenin signaling is indispensable for PPARγ induction and preadipocyte differentiation [40]. Interestingly, we observed that SAOS-2 cells treated with the OATS and then cultured in an adipogenic differentiation medium seemed to be driven towards an adipogenic profile. These cells showed an increasing expression of the adipogenic markers PPARɣ and CEBP-α as well as perlipin, a structural coat protein found on intracellular lipid droplets that is usually associated with adipogenesis. PPARγ was reported to enhance perilipin gene expression, resulting in lipid droplets storing triacylglycerol in adipocytes [41].

Our findings indicate that the secretion from the adipose tissue of individuals with obesity has a detrimental impact on osteoblast maturation that may favor adipogenic differentiation. Thus, it is conceivable to assume that an obese adipose microenvironment contributes to a regression in the osteoblast phenotype, potentially leading to osteoblast dedifferentiation and promoting a shift towards an adipocyte-like behavior. Noteworthy, the ability of the OATS to promote the transdifferentiation of osteoblasts may contribute to the presence of enriched lipid cells in the bone marrow, which are usually observed in individuals with obesity [42]. The association between increased lipid droplet accumulation in osteoblasts and/or osteocytes and the concurrent expansion of bone marrow adipose tissue have often been correlated with a low bone mass [42].

The adipose tissue, particularly visceral adipose tissue (AT), stands out as a significant source of cytokines and growth factors in the context of obesity. This includes the release of TNFα, MIP1α, IL-6, IL10, TGF-β, and VEGF [43], which have been implicated in contributing to the development of low-grade systemic inflammation. This inflammatory milieu has the potential to disrupt the homeostasis of various cell systems [18]. Infiltrated immune cells in AT are key contributors to the secretion of these mediators, altering the homeostasis of the adipose microenvironment and consequently affecting the fate and behavior of other AT resident cells.

We conducted a comparative analysis of the adipokines in the secretomes of subcutaneous adipose tissue (AT) from lean and obese individuals. In contrast to our observations in visceral AT depots [43], the analysis, even considering the variability among samples, did not detect significant differences between the contents of pro-inflammatory adipokines in the secretomes of lean vs. obese subcutaneous AT. However, the concentration of TGF-β1 was notably increased in the OATS.

TGF-β family members are reported to exhibit a dual role in regulating osteoblast differentiation. While some of these members can promote osteoblast maturation, under certain conditions, a high or prolonged exposure to TGF-β1 can inhibit osteoblast differentiation. These effects of TGF-β1 on osteoblasts are highly context-dependent, and may vary depending on factors such as the concentration, the time of exposure, and the presence of other signaling molecules. The dual role of TGF-β1 in bone remodeling reflects its complex and intricate involvement in the regulation of osteoblast functions [44,45,46,47]. Supporting our data, other studies have shown that TGF-β inhibits mineralization in pre-osteoblasts [44,45,46,47], and may induce changes in cell shape towards a mesenchymal conformation along with a reduction in alkaline phosphatase activity and osteogenic marker expression [45,47]. We showed that TGF-β1 mimicked most effects of the OATS on SAOS-2 cells, interfering with viability, proliferation, and morphology. Additionally, TGF-β1 inhibited extracellular matrix calcification and decreased the expression of osteogenic markers in osteoblasts. The results point to TGF-β1 as an important player in osteoblast dedifferentiation triggered by obese subcutaneous AT secretion. Confirming this premise, the blocking of TGF-βR activity by LY210961, a pan-receptor antagonist, was capable of partially inhibiting the negative effects caused by the OATS on osteoblast differentiation, also impairing the increase in the expression of PPAR-ɣ, a known adipogenic marker.

This study has some limitations that should be addressed. Although our data evidenced a significant role of TGF-β1 in the OATS effects on osteoblastogenesis, we cannot discard the effects of the other adipokines present in the secretome from the subcutaneous adipose tissue during obesity. Despite the fact that these other adipokines were not detected at significant levels, they may act in concert to interfere with osteoblastic differentiation. Two of them called attention: leptin, which showed a tendency to increase, and TGF-β3, which tended to be lower in the OATS, possibly through a compensatory mechanism to increase TGF- β1. Both could potentially contribute to the effects observed, and their roles should be studied further to understand their individual and combined impacts on osteogenesis. Moreover, the substantial enrichment in the free fatty acids of the OATS is another point that should be considered. Therefore, it is conceivable to suggest that the increased concentration of free fatty acids and TGF-β1 in the OATS may possibly be the important drivers involved in the transdifferentiation of osteoblasts into adipocyte-like cells in obesity.

## 5. Conclusions

Our research demonstrates that the adipose tissue secretome of obese individuals significantly impacts osteoblast function and behavior. This impact includes the modulation of key signaling pathways and the altered expression of differentiation markers, ultimately leading to the dedifferentiation of osteoblasts. These cells regress from a mature state to an osteoprogenitor-like profile and may even transdifferentiate into adipocyte-like cells. Notably, TGF-β1 plays a crucial role in these processes. These findings reveal a novel mechanism by which the interaction between adipose tissue and bone cells contributes to osteoporosis in obese individuals, offering fresh insights into the complex dynamics between obesity and bone health.

## Figures and Tables

**Table 1:** Characteristics of the patients enrolled in the study.

Group	Samples	Age	BMI	Female	Male *
Lean	9	36.2 ± 8.8	23.4 ± 2.3	8	1
With Obesity	10	43.2 ± 6.3	45.2 ± 6.9	9	1

Data of patients used in the study. BMI: body mass index. * Bias was minimized by the inclusion of one male donor in each group; the selected subjects were of a similar age (35 ± 1.5) and their clinical profiles were not divergent from the other female participants inside their respective groups.

## Data Availability

The data that support the findings of this study are openly available on the repository Figshare at http://doi.org/10.6084/m9.figshare.24806004, reference number [https://figshare.com/s/de2a9d6d93b44f49d0ec]. Accessed on 25 April 2024.

## Supplementary Materials

The following supporting information can be downloaded at: https://www.mdpi.com/article/10.3390/cells13171418/s1, Figure S1: Obese secretome effects on osteoblasts viability and proliferation in the absence of FBS; Figure S2: Release of proinflammatory adipokines and free fatty acids by subcutaneous adipose tissue in vitro; Figure S3: TGF- β1 reproduces partially OATS effects.
