# Supplementary figures and images for: Unlocking the Secrets of Adipose Tissue: How an Obesity-Associated Secretome Promotes Osteoblast Dedifferentiation via TGF-β1 Signaling, Paving the Path to an Adipogenic Phenotype
Source: Cells. 2024 Aug 25;13(17):1418. doi: 10.3390/cells13171418 (PMC11394205; doi:10.3390/cells13171418)

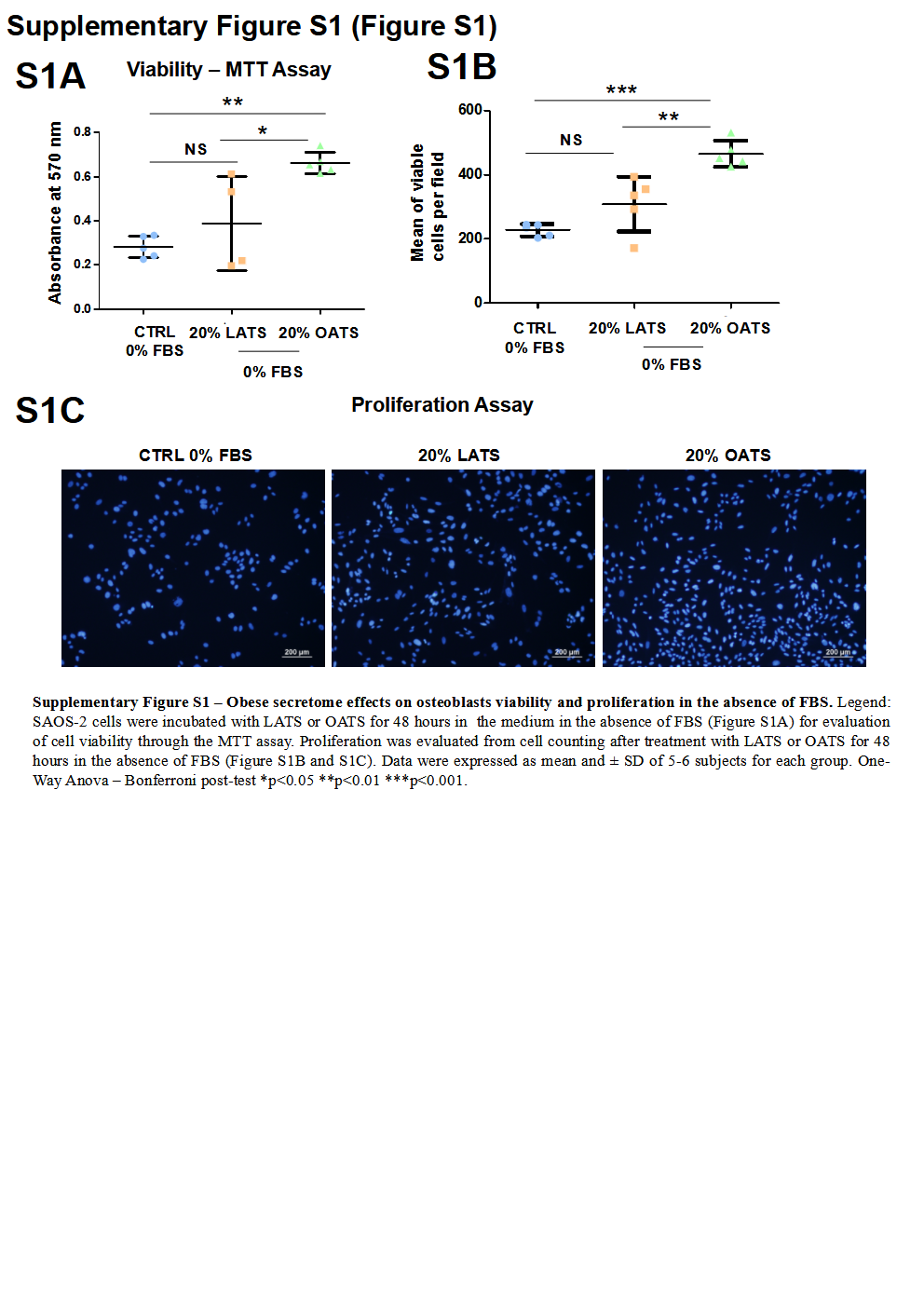

Supplement: Supplementary file 1 [file cells-13-01418-s001.zip › SUPPLEMENTARY FIGURE S1.tif]

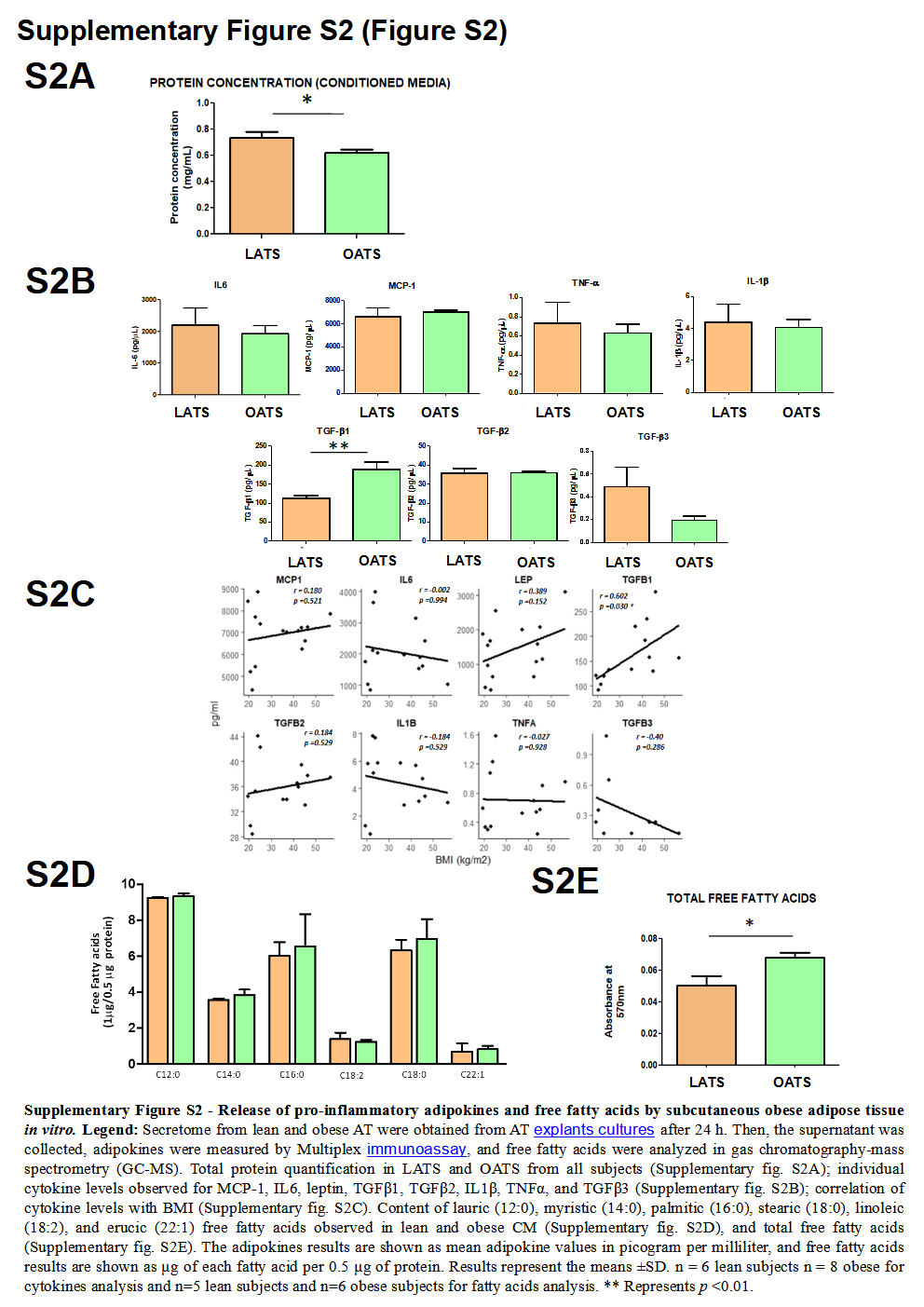

Supplement: Supplementary file 1 [file cells-13-01418-s001.zip › SUPPLEMENTARY FIGURE S2.tif]

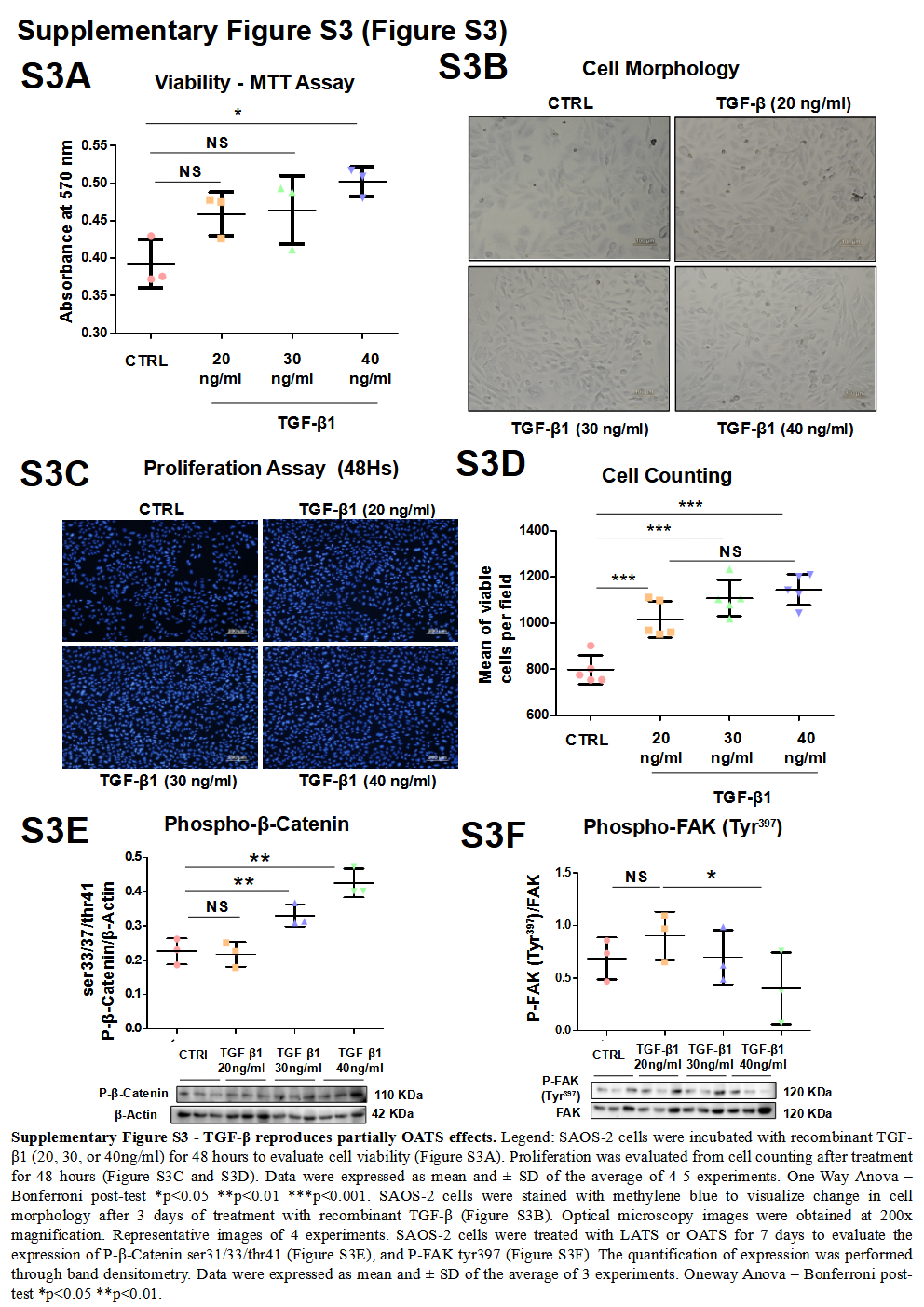

Supplement: Supplementary file 1 [file cells-13-01418-s001.zip › SUPPLEMENTARY FIGURE S3.tif]
